# Supplementary material for: DeepSTABp: A Deep Learning Approach for the Prediction of Thermal Protein Stability
Source: Int J Mol Sci. 2023 Apr 18;24(8):7444. doi: 10.3390/ijms24087444 (PMC10138888; doi:10.3390/ijms24087444)
Supplement: Supplementary file 1 [file ijms-24-07444-s001.zip › ijms-2327586-supplementary.pdf]

DeepSTABp: A Deep Learning Approach for the Prediction of Thermal Protein Stability

Supplementary Information

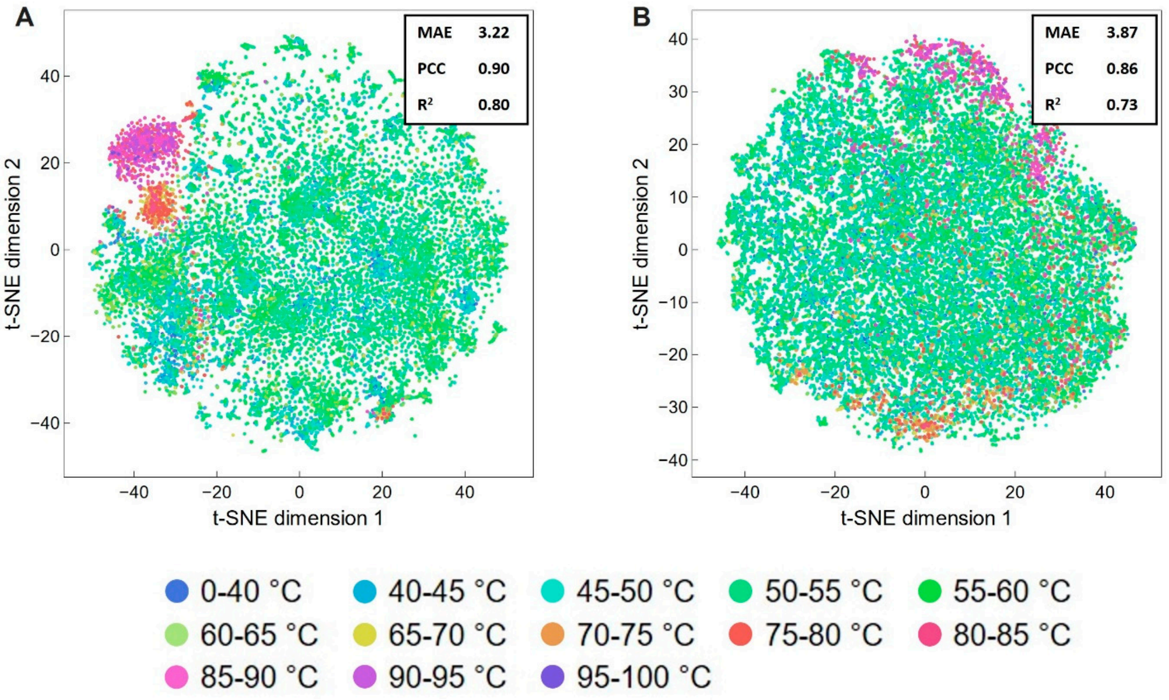

Figure S1. Comparison of Amino acid sequence embedding using the protein language model ProtT5-XL-UniRef50 (A) and protr feature extraction used by ProtStab2 (B). To compare the effectiveness of using a protein language model versus traditional protein feature extraction methods for protein sequence embedding, we replaced the embedding generated by the transformer block of the final DeepSTABp model with an embedding created using classical feature extraction methods. The features were acquired using the protr package and were chosen according to the recommendations of the ProTstab2 authors, factoring in the performed feature selection. By visualizing the activations of each individual block, we observed that the protein language model for sequence embedding allows for t-SNE to distinguish proteins that remain stable at high temperatures (orange and purple), which is not possible using the feature-based embedding computed by protr. These results are reflected when computing performance metrices on the testing dataset, which indicate that the model utilizing the protein language model for protein sequence embedding (**A**) significantly outperforms the model utilizing classical feature extraction methods (**B**).
